# Evaluation of the London Measure of Unplanned Pregnancy (LMUP) among a nationally representative sample of pregnant and postpartum women Ethiopia^☆^^☆☆^

**DOI:** 10.1016/j.conx.2023.100094

**Published:** 2023-04-05

**Authors:** Celia Karp, Caroline Moreau, Solomon Shiferaw, Assefa Seme, Mahari Yihdego, Linnea A. Zimmerman

**Affiliations:** a Department of Population, Family and Reproductive Health, Johns Hopkins Bloomberg School of Public Health, Baltimore, MD, United States; b Soins et Santé Primaire, CESP Centre for Research in Epidemiology and Population Health U1018, Inserm, F-94805 Villejuif, France; c Department of Reproductive Health and Health Service Management, School of Public Health, Addis Ababa University, Addis Ababa, Ethiopia

**Keywords:** Ethiopia, Measurement, Pregnancy preferences, Sub-Saharan Africa, Unintended pregnancy

## Abstract

**Objectives:**

Rigorous measurement of pregnancy preferences is needed to address reproductive health needs. The London Measure of Unplanned Pregnancy (LMUP), developed in the UK, has been adapted for low-income countries. Psychometric properties of LMUP items remain uncertain in contexts with limited access to and use of health services.

**Study design:**

This cross-sectional study examines the six-item LMUP’s psychometric properties among a nationally representative sample of 2855 pregnant and postpartum women in Ethiopia. Principal components analysis (PCA) and confirmatory factor analysis (CFA) estimated psychometric properties. Hypothesis testing examined associations between the LMUP and other measurement approaches of pregnancy preferences using descriptive statistics and linear regression.

**Results:**

The six-item LMUP had acceptable reliability (α = 0.77); two behavioral items (contraception, preconception care) were poorly correlated with the total scale. A four-item measure demonstrated higher reliability (α = 0.90). Construct validity via PCA and CFA indicated the four-item LMUP’s unidimensionality and good model fit; all hypotheses related to the four-item LMUP and other measurement approaches were met.

**Conclusions:**

Measurement of women’s pregnancy planning in Ethiopia may be improved through use of a four-item version of the LMUP scale. This measurement approach can inform family planning services to better align with women’s reproductive goals.

**Implications:**

Improved pregnancy preference measures are needed to understand reproductive health needs. A four-item version of the LMUP is highly reliable in Ethiopia, offering a robust and concise metric for assessing women’s orientations toward a current or recent pregnancy and tailoring care to support them in achieving their reproductive goals.

## 1 Introduction

Measurement of unintended pregnancy remains a long-standing focus of the reproductive health field. Globally, nearly half of all pregnancies are categorized as “unintended”, either as unwanted or mistimed [1]. This retrospective, timing-based measure, defined by asking women whether a pregnancy occurred at the right time, is often critiqued for oversimplifying a complex and multifaceted latent construct [2], [3], [4]. Researchers emphasize that not all women formulate concrete intentions and many adapt their intentions to their social and economic circumstances [5], [6], [7], [8].

Over the last two decades, researchers advanced the measurement of pregnancy preferences—or women’s reactions toward a pregnancy—by accounting for affective, behavioral, and timing-based dimensions. Aiken and colleagues proposed a new framework for measuring “pregnancy acceptability,” recognizing that not all unintended pregnancies are experienced as adverse events [2]. This concept of acceptability offers new and more nuanced ways for understanding pregnancies that have historically been categorized as “unintended.” In a similar effort to offer a more nuanced measure about women’s perspective on pregnancy, the retrospective London Measure of Unplanned Pregnancy (LMUP), developed in the UK, uses a multifaceted approach, integrating women’s self-reported pregnancy-related behaviors (e.g., preconception care, contraceptive use), context (e.g., timing, intention, wantedness), and couple communication about childbearing [9]. The six-item LMUP is a psychometrically robust measure, which offers insights into understudied, critical dimensions of women’s pregnancy preferences, such as women’s emotional response toward the pregnancy and partner engagement in reproductive decision-making, and related health outcomes.

Since its development, the LMUP was adapted into an interviewer-administered format for use among populations with low literacy [10] and translated into 16 languages, including use in six sub-Saharan African countries: Kenya [11], Malawi [12], [13], Mozambique [14], Sierra Leone [15], Uganda [16], and Ethiopia [17]. While psychometric properties of the LMUP are promising, with acceptable-to-high internal consistency reliability in several African settings [10], [15], [17], multiple studies identified challenges related to items reflecting preconception behaviors [10], [15], [17], [18]. Likewise, a small, facility-based study among women seeking antenatal care (ANC) in Ethiopia reported acceptable reliability and validity of the LMUP but noted concerns related to specific items reflecting pre-pregnancy behaviors and contraceptive use [17]. In addition to concerns related to the salience of specific items, reliance on specific populations, such as those receiving ANC, may not be generalizable, as individuals who seek care may be more positively oriented toward their pregnancy and have greater access to resources than the general population. The extent to which the LMUP has relevance and validity among a broader sample of women in Ethiopia, including those who may not seek ANC, is unknown, limiting understanding of the LMUP’s reliability and validity in contexts with limited access to and use of health services.

This study aims to examine the psychometric properties of the LMUP among a nationally representative sample of pregnant and postpartum women in Ethiopia, and assess construct validity of the LMUP by examining how LMUP scores relate to other widely used items measuring pregnancy preferences.

## 2 Methods

### 2.1 Data collection

We use data from Performance Monitoring for Action (PMA) Ethiopia, a collaboration between Addis Ababa University, the Ethiopian Federal Ministry of Health, and Johns Hopkins Bloomberg School of Public Health. A household census was conducted in 217 enumeration areas across six regions, representing 90% of Ethiopia’s population, between October and November 2019. Women ages 15–49 were eligible if they were pregnant or within 6 weeks postpartum at screening. Altogether, 2855 consenting women were enrolled in the survey and completed a baseline interview (response rate = 99.6%). Study procedures were approved by Addis Ababa University [075/13/SPH] and Johns Hopkins Bloomberg School of Public Health [FWA00000287] Institutional Review Boards. Additional information about PMA Ethiopia is described elsewhere [19].

### 2.2 Measures

Our four measures of pregnancy preferences included the LMUP scale, a timing-based question, women’s emotional response to their pregnancy, and an intersectional measure reflecting the correspondence between timing-based and emotional responses. The six-item Chichewa version of the LMUP, adapted for use in Malawi, was used due to contextual similarities in access to and use of health services relevant for items 1 (contraception) and 6 (preconception preparation) (Table 1) [10]. As the first application of the LMUP among a nationally representative sample of pregnant and postpartum women in Ethiopia, we conducted cognitive testing of each item, translated into Amharic, Tigrinya, and Afan Oromo, among five women seeking ANC; no wording changes were deemed necessary.

**Table 1:** Distribution of LMUP item responses and missingness, N = 2855

**Item and response options**	**Freq**	**%**
**Item 1 (Contraception)**		
*In the month that you became pregnant, you:*		
2) not using contraception	2614	91.6
1) were using contraception, but not on every occasion	93	3.3
1) always used contraception, but knew that the method had failed at least once	12	0.4
0) always used contraception	110	3.8
Missing	25	0.9
**Item 2 (Timing)**		
*In terms of becoming a mother (first time or again), you feel that your pregnancy happened at the:*		
2) Right time	1802	63.1
1) OK, but not quite right time	557	19.5
0) Wrong time	491	17.2
Missing	5	0.2
**Item 3 (Intention)**		
*Just before you became pregnant:*		
2) You intended to get pregnant	1826	63.7
1) Your intentions kept changing	240	8.4
0) You did not intend to get pregnant	784	27.4
Missing	6	0.2
**Item 4 (Desire)**		
*Just before you became pregnant:*		
2) You wanted to have a baby	2043	71.6
1) You had mixed feelings about having a baby	251	8.8
0) You did not want to have a baby	559	19.6
Missing	3	0.1
**Item 5 (Partner)**		
*Before you became pregnant, you and your partner:*		
2) Agreed for you to get pregnant	1548	54.0
1) Discussed having children together, but hadn’t agreed for you to get pregnant	373	13.0
0) Never discussed having children together	925	32.3
Missing	9	0.3
**Item 6 (Preparation)**		
*Before you became pregnant, did you do any of the following in preparation for pregnancy: took folic acid/vitamins; ate more healthily; sought medical/health advice; saved money for healthcare; did none of the above.*		
2) Did two or more preparatory lifestyle changes	137	4.8
1) Did one preparatory lifestyle changes	254	8.9
0) No preparatory lifestyle changes	2439	85.4
Missing	25	0.8

Notes: LMUP, London Measure of Unplanned Pregnancy.

Amharic, Tigrinya, and Afan Oromo versions of the LMUP adapted from the Chichewa version of the LMUP due to contextual similarities in access to and use of health services, relating to items 1 (contraception) and 6 (preparation), across geographies.

Each LMUP item was scored on a range of 0–2, following guidance provided by Barrett and colleagues [9]. Continuous and categorical variables were constructed using responses to the LMUP items, with the continuous variable (total score: 0–12) reflecting a sum of responses to six items, as recommended [20]. The categorical measure grouped women according to their total score: 0–3 (unplanned), 4–9 (ambivalent), 10–12 (planned or highly planned). Women’s timing-based intentions were assessed as a categorical variable by asking women, “At the time you became pregnant, did you want to become pregnant then, later, or not at all?”. Women who responded “later” or “not at all” were categorized as experiencing an “unintended pregnancy”. This timing-based question is widely used in the reproductive health field as a measure of unintended pregnancy, including by the Demographic and Health Surveys program [21]. Emotional response to a pregnancy was ascertained by asking women how they felt when they found out they were pregnant. Response options ranged from “very happy” to “very unhappy” and were grouped into a categorical variable: happy (including very happy), ambivalent, and unhappy (including very unhappy). The question was included in the PMA survey to expand measurement of pregnancy preferences beyond the timing-based question and to capture women’s affective reactions toward a pregnancy [2]. Similar measures have been fielded by researchers among women in Malawi [8] and in the United States [22], [23], [24]. Finally, to capture the intersection between timing-based intentions and feelings toward a pregnancy, we grouped women into one of four pregnancy acceptability categories: happy and wanted; mixed feelings with greater acceptability; mixed feelings with lower acceptability; or unhappy and unwanted (Table 2). This intersectional variable was included to investigate associations between women’s pregnancy “planning”, as measured by the LMUP, and how “acceptable” a pregnancy was in terms of the timing and emotional response.

**Table 2:** Categorization of the intersectional measure of timing-based and emotional responses to a pregnancy

Sociodemographic characteristics included age (15–24, 25–34, 35–49 years), marital status (married/in-union vs unmarried/not in-union), residence (urban vs rural), household wealth quintile, education (none, primary, secondary or higher), and region. Reproductive characteristics included parity (0, 1, 2, 3 or more children), pregnancy status at baseline (pregnant vs postpartum), and contraceptive ever use (yes vs no).

### 2.3 Analytic sample

Our primary analytic sample included all women who were pregnant or within 9 weeks postpartum at baseline (n = 2855). Analyses of the LMUP score were restricted to the 2781 women with complete data on all questions on pregnancy preferences (97%).

### 2.4 Analysis

We used classical test theory to examine how the LMUP scale and item properties functioned, replicating processes used for the measure’s development and subsequent validations [10], [14], [16], [17], [25], [26], [27]. Item acceptability was assessed based on LMUP item missingness, with<5% missingness indicating greater item acceptability. Assessment of item discrimination indices included calculating item-rest correlations (i.e., correlation of each item with the total score of the remaining LMUP items), with a minimum acceptable cutoff of 0.2, and item-test correlations (i.e., correlation of each item with the total score), which were examined to confirm all items were positively correlated [28], [29]. Internal consistency reliability was assessed using a Cronbach’s alpha (α) statistic with a pre-set acceptable minimum of α > 0.7 [30]. Next, to assess construct validity (i.e., the degree to which the LMUP scores correlated with other variables, as hypothesized), we used principal components analysis (PCA) and hypothesis testing. PCA was used to confirm the scale’s unidimensionality (i.e., all items reflected one construct), with all items loading> 0.4 to one component with an eigenvalue >1. Confirmatory factor analysis (CFA) was also used to assess structural validity, via factor loadings>0.4, and assess acceptable model fit, determined through assessment of the comparative factor index (CFI > 0.95) and standardized root mean squared residual (SRMR < 0.08) [31], [32]. Sensitivity analyses were conducted by independently removing items with poor functioning and repeating CFA on the refined item set. Abbreviated measures were compared against the six-item LMUP scale using summary statistics. Hypothesis testing was grounded in the literature and evaluated whether LMUP scores were lower (i.e., pregnancies were *less* planned) among women who 1) were unhappy to learn they were pregnant (per the emotional response question); 2) experienced an unintended pregnancy (per the timing-based question); 3) had pregnancies categorized as “unhappy and unwanted” (per the intersectional variable), and 4) had three or more children [10], [22], [24]. Weighted ranges and mean scores of the LMUP scale were calculated for each categorical variable. Statistical significance was assessed using linear regression models, with *p* < 0.05 considered statistically significant. Sensitivity analysis was conducted to assess if LMUP scores or psychometric properties varied by pregnancy status at time of enrollment, as postpartum women may be more likely to report pregnancies as planned or wanted, regardless of their preferences when they learned they were pregnant. Analyses used survey weights to adjust for the complex survey design and were conducted using Stata 16.

## 3 Results

### 3.1 Sample characteristics

Table 3 presents sociodemographic and reproductive characteristics of women. The sample was predominantly rural, with primary or no education, married, and multiparous. Most (78.4%) were pregnant at the time of the survey. About one-third of pregnancies were classified as unintended, per the timing-based question, and 31.1% of women were either ambivalent or unhappy when they learned they were pregnant, according to the emotional response question.

**Table 3:** Characteristics of a nationally representative sample of women aged 15–49 years who are currently pregnant or within 6 weeks postpartum in Ethiopia (N = 2855), 2019

**Characteristic**	**%**	**N**
Age (mean years)	27.3	
Currently married	97.7	2790
Living in urban area	22.3	637
Wealth		
Lowest	20.1	577
Lower	20.0	568
Middle	19.9	571
Higher	20.1	574
Highest	19.9	565
Education		
None	41.2	1180
Primary	40.2	1145
Secondary or higher	18.7	530
Region		
Tigray	7.0	200
Afar	2.0	57
Amhara	20.2	577
Oromiya	43.9	1253
SNNP	23.1	658
Addis	3.9	110
Number of children		
Nulliparous	18.7	534
1	21.3	609
2	15.2	435
3 or more	44.6	1274
Pregnancy status at baseline		
Pregnant	78.4	2239
0–4 weeks postpartum	11.4	328
5–9 weeks postpartum	10.1	288
Contraceptive history		
Ever used a method	61.0	1742
Timing-based pregnancy intention^a^		
Wanted then	63.7	1818
Wanted later	27.1	774
Did not want at all	9.0	258
Emotional response toward pregnancy^a^		
Happy or very happy	68.5	1954
Ambivalent (mixed)	15.3	438
Unhappy or very unhappy	15.9	454
Pregnancy acceptability^a^		
Happy and intended	58.1	1661
Mixed with greater acceptability	14.6	415
Mixed with lower acceptability	12.1	345
Unhappy and unintended	14.8	423

Notes: Weighted counts and percentages.

a Missing<0.5% (n < 15) of observations.

### 3.2 Descriptive analysis and missingness

**Fig. 1:**
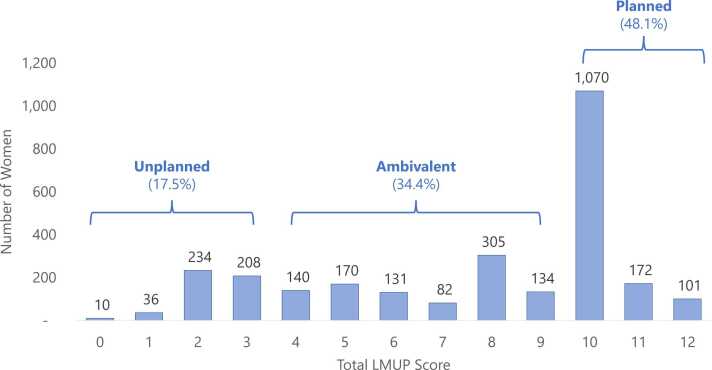
Distribution of 6-Item London Measure of Unplanned Pregnancy (LMUP) scores among pregnant and postpartum women in Ethiopia, 2019 (N = 2781).

Table 3 describes distributions of responses and missingness by LMUP item. Missingness was very low (<1%) for all items and was highest for the contraception item (0.9%). Figure 1 displays the distribution of the total LMUP score, illustrating a left skew of responses with a full score range from 0 to 12 and mean score of 7.6 (sd = 3.15). Altogether, 17.5% of women were categorized as experiencing pregnancies that were unplanned (score 0–3), 34.4% as ambivalent (score 4–9), and 48.1% as planned (score 10–12).

### 3.3 Item and scale properties: reliability, PCA, and CFA

Table 4 presents item and scale properties, including construct validity results. The six-item LMUP scale had acceptable reliability (Cronbach alpha [α] = 0.77). Item-test correlations were positive for all items, while two behavioral items, reflecting contraceptive use and preconception care, were poorly correlated with the full scale (item-rest: 0.18 and 0.16, respectively). PCA indicated the six-item LMUP scale loaded onto one component (eigenvalue = 3.51), with evidence of a second component (eigenvalue = 0.99). The two behavioral items also loaded below the minimum threshold onto the first component (contraception = 0.15 and preconception care = 0.14). Sensitivity analyses, independently removing the behavioral items and rerunning analyses, improved scale reliability (α = 0.83–0.84). Exclusion of the partner discussion item increased scale reliability slightly (α = 0.92) but was retained due to acceptable correlations and factor loadings. Given item and scale properties, we dropped the two behavioral items, resulting in an abbreviated, four-item LMUP scale with high reliability (range: 0–8; α = 0.90). CFA of the four-item LMUP showed good model fit (CFI = 0.99, SRMR = 0.03; data not shown), with all factor loadings>0.4; improving upon the initial CFA of the six-item measure.

**Table 4:** Initial and final Principal Components Analysis (PCA) and Confirmatory Factor Analysis (CFA) of London Measure of Unplanned Pregnancy (LMUP), N = 2781

**Item**	**Item-test**	**Item-rest**	**PCA component loadings**	**CFA factor loadings**	**Excluded item Alpha**
**Initial Confirmatory Factor Analysis (6 items)**					
1. Contraception	0.41	0.18^a^	0.15^a^	0.20^a^	0.83
2. Timing	0.85	0.75	0.51	0.88	0.72
3. Intention	0.88	0.80	0.52	0.92	0.70
4. Desire	0.82	0.71	0.49	0.83	0.73
5. Partner	0.74	0.59	0.42	0.66	0.76
6. Preparation	0.40	0.16^a^	0.14^a^	0.18^a^	0.84
			**Initial 6-item LMUP Alpha**		0.77
**Final Confirmatory Factor Analysis (4 items)**					
1. Contraception	-	-	-	-	-
2. Timing	0.90	0.81	0.52	0.87	0.85
3. Intention	0.93	0.87	0.54	0.93	0.83
4. Desire	0.88	0.78	0.51	0.83	0.86
5. Partner	0.78	0.62	0.44	0.65	0.92
6. Preparation	-	-	-	-	-
		**Abbreviated 4-item LMUP Alpha**			0.90

- Indicates item excluded.

a Values below minimum acceptable criteria.

### 3.4 Construct validity hypothesis testing

Table 5 presents results of the construct validity hypothesis tests, demonstrating that all four hypotheses were met with the abbreviated four-item LMUP. Specifically, the four-item LMUP scores were lower among: women who were unhappy to learn they were pregnant, relative to those who were happy/very happy (*p* < 0.001); women who experienced a pregnancy they did not want, relative to those whose pregnancies were wanted (*p* < 0.001); women who were unhappy about their pregnancy and for whom it was unintended, relative to those who were happy and intended to become pregnant (*p* < 0.001); and women with three or more children, relative to women with fewer children (*p* < 0.001). Descriptive and psychometric results did not differ according to women’s pregnancy status (pregnant vs postpartum).

**Table 5:** Construct validity hypothesis tests of London Measure of Unplanned Pregnancy (LMUP) abbreviated 4-item measure, N = 2781

**Hypothesis**		**Variable**	**LMUP 4-item score range (mean)**	***p*-value**
**LMUP scores will be lower among…**				
	women who were unhappy to learn they were pregnant, relative to those who were happy.	**Emotional response**		
		Unhappy or very unhappy	0–8 (1.54)	<0.001*
		Ambivalent	0–8 (3.22)	
		Happy or very happy	0–8 (7.02)	
	women who experienced a pregnancy they did not want at all, relative to those whose pregnancies were wanted then.	**Timing-based measure**		
		Did not want at all	0–8 (1.21)	<0.0001*
		Wanted later	0–8 (2.87)	
		Wanted then	0–8 (7.33)	
	women who were both unhappy about their pregnancy and for whom the pregnancy was unintended, relative to those who were happy and for whom the pregnancy was intended.	Intersectional measure		
		Unhappy and unintended	0–8 (1.35)	<0.001*
		Mixed feelings with lower acceptability	0–8 (2.55)	
		Mixed feelings with greater acceptability	0–8 (4.49)	
		Happy and intended	0–8 (7.53)	
	women with three or more children, relative to women with fewer children.	Number of children		
		0	0–8 (6.64)	<0.001*
		1	0–8 (6.18)	
		2	0–8 (5.64)	
		3 or more	0–8 (4.71)	

* p-value from bivariable linear regression models.

## 4 Discussion

We examined the psychometric properties of the six-item version of the LMUP among a nationally representative sample of pregnant and postpartum women in Ethiopia. Findings indicated a high level of item acceptability, with fewer than 1% of missing responses for all items, but poor correlations with the total scale score and low component and factor loadings of two items—contraceptive use and preconception preparation—which fell below acceptable, psychometric standards for inclusion in the measure. An abbreviated, four-item measure demonstrated high reliability and construct validity and was highly correlated with other measurement approaches of pregnancy preferences.

Results underscore the importance of accounting for contextual differences that can hinder the transferability of measures across diverse settings. As observed in Ethiopia [17] and other contexts, including Malawi [12], Sierra Leone [15], and India [18], poor functioning of two preconception behavioral items in our study may reflect differences in women’s access to and use of maternal and reproductive health services, thereby limiting the utility of these measures as a marker of individuals' pregnancy preferences. Fewer than one in five women in our sample reported engaging in any preconception practice (e.g., taking folic acid supplementation, seeking medical advice). While Ethiopia’s coverage of pregnancy-related services has grown considerably in the last decade, recent data indicate that only 12% of women receive counseling on iron and folate supplementation, suggesting preconception care remains low [33]. Similarly, few women in our sample reported consistently using contraception at the time they became pregnant (<5%), despite wide variation in other dimensions of pregnancy planning, according to the LMUP. Nationally, 36% of married women use modern contraception, and many wait until after their second child to begin using a method, particularly in rural areas [34]. The low correlations, component, and factor loadings of the contraception item observed in our study are comparable with findings among a small, facility-based sample of women in Ethiopia, in which the item was negatively correlated with all other LMUP items [17]. Given these patterns, it is not surprising that women’s preconception behaviors—including use of contraception—were misaligned with other aspects of their pregnancy preferences. The utility of these items for capturing elements of pregnancy planning remains uncertain among populations with limited access to or use of health services. Such inconsistencies warrant further investigation to ensure the measurement of pregnancy preferences reflects the realities of women’s circumstances in these geographies.

To our knowledge, this is the first study to apply an iterative approach to evaluating the LMUP by examining an abbreviated, four-item version of the scale that addresses psychometric weakness of the six-item measure in our study context. Our finding that the four-item LMUP was, psychometrically, a more robust measure than the full, six-item measure—determined via reliability, PCA, and CFA—without limiting the measure’s construct validity—assessed through its’ association with other measurement approaches of pregnancy preferences—is encouraging. In new contexts where the LMUP is tested, excluding items that lack psychometric standards for inclusion may enable the integration of a shorter, more concise version of the LMUP for future research, while enhancing the psychometric properties of the measure itself. We echo recommendations of other scholars that qualitative research should be used to identify opportunities for improving the LMUP and its alignment with women’s conceptualization of pregnancy preferences in geographies where the behavioral items prove challenging [18].

The abbreviated LMUP holds promise in capturing nuance of women’s pregnancy perspectives. Results of hypothesis testing with the timing-based and emotional response questions, and a variable reflecting the intersection of these dimensions were consistent: women who were unhappy, those who experienced unwanted pregnancies, and those reporting both reactions had the lowest LMUP scores. An enhanced focus on pregnancy acceptability, including through the four-item LMUP and other emerging measures of pregnancy preferences, can better reflect the complexity of social, economic, and health circumstances that shape women’s perceptions of pregnancy. The inclusion of items capturing women’s pregnancy desires, intentions, preferred timing, and partner engagement in the four-item LMUP may better serve the conceptual shift from the timing-based paradigm toward a more comprehensive understanding of the acceptability of a pregnancy within individuals’ contexts [2]. Additionally, the four-item LMUP's use may reduce bias introduced by the over-simplification of pregnancies as intended or unintended and strengthen understanding of the relationship between pregnancy preferences and health outcomes [13]. This measurement approach can be used to inform family planning services by enabling practitioners and researchers to ascertain women’s pregnancy preferences more accurately and support them in achieving their reproductive goals.

We acknowledge study limitations, including the lack of extensive qualitative research to understand potential gaps in content of the LMUP among women in Ethiopia. We implemented pilot-testing of LMUP items to ensure item-specific comprehension but recognize the appropriateness of the items should be evaluated in more in-depth qualitative work. Additionally, we administered the LMUP within an existing cohort study of pregnant and postpartum women who were asked to respond to LMUP items only once, thereby limiting our ability to evaluate test-retest reliability over 2 weeks, as recommended for psychometric evaluation [28]. Finally, given the parent study, we were unable to evaluate the predictive utility of the LMUP with pregnancy outcomes, such as abortion, as has been done elsewhere [35]. This sampling design resulted in an underrepresentation of individuals whose pregnancies ended in abortion and for whom responses on the LMUP items may vary considerably from women who continued their pregnancies.

Despite these limitations, this study offers novel insights into the potential of an abbreviated, four-item version of the LMUP for use in Ethiopia and similar sub-Saharan African geographies. Future research should longitudinally explore how each measure of pregnancy preferences, including the four-item LMUP, operates in relation to reproductive health outcomes to determine which measure, or combination of measures, is most relevant for shaping maternal, reproductive, and child health outcomes.

## 5 **Conclusion**

An abbreviated, four-item version of the LMUP scale is reliable and valid in the Ethiopian context. Researchers and programs using the LMUP in Ethiopia and similar sub-Saharan African settings with limited preconception care and contraceptive use may consider removing the two pregnancy-related behavioral items to explore retrospective pregnancy preferences. Future adaptions of the LMUP or other measures of pregnancy preferences in new contexts, particularly those in financially-constrained settings, would benefit from qualitative research to contextualize measurement within women’s reproductive circumstances.
